# Unsupervised progressive elastic band exercises for frail geriatric inpatients objectively monitored by new exercise-integrated technology—a feasibility trial with an embedded qualitative study

**DOI:** 10.1186/s40814-017-0202-3

**Published:** 2017-11-13

**Authors:** C. R. Rathleff, T. Bandholm, E. G. Spaich, M. Jorgensen, J. Andreasen

**Affiliations:** 1 Department of Health Science and Technology, Aalborg University, Fredrik Bajers Vej 7-D3, 9220 Aalborg, Denmark; 2 Department of Physiotherapy and Occupational Therapy, Aalborg University Hospital, Hobrovej 18-22, 9000 Aalborg, Denmark; 3 Physical Medicine & Rehabilitation Research - Copenhagen (PMR-C), Department of Physical and Occupational Therapy, Department of Orthopedic Surgery, Clinical Research Center, Amager-Hvidovre Hospital, University of Copenhagen, Copenhagen, Denmark; 4 Center for PREdiction and prevention of FALLs (PREFALL) Department of Geriatrics, Aalborg University Hospital, Hobrovej 18-22, Aalborg, Denmark

**Keywords:** Frail elderly, Elastic band exercises, Unsupervised exercises, Monitoring technology, BandCizer, Adherence, Feasibility

## Abstract

**Background:**

Frailty is a serious condition frequently present in geriatric inpatients that potentially causes serious adverse events. Strength training is acknowledged as a means of preventing or delaying frailty and loss of function in these patients. However, limited hospital resources challenge the amount of supervised training, and unsupervised training could possibly supplement supervised training thereby increasing the total exercise dose during admission. A new valid and reliable technology, the BandCizer, objectively measures the exact training dosage performed. The purpose was to investigate feasibility and acceptability of an unsupervised progressive strength training intervention monitored by BandCizer for frail geriatric inpatients.

**Methods:**

This feasibility trial included 15 frail inpatients at a geriatric ward. At hospitalization, the patients were prescribed two elastic band exercises to be performed unsupervised once daily. A BandCizer Datalogger enabling measurement of the number of sets, repetitions, and time-under-tension was attached to the elastic band. The patients were instructed in performing strength training: 3 sets of 10 repetitions (10–12 repetition maximum (RM)) with a separation of 2-min pauses and a time-under-tension of 8 s. The feasibility criterion for the unsupervised progressive exercises was that 33% of the recommended number of sets would be performed by at least 30% of patients. In addition, patients and staff were interviewed about their experiences with the intervention.

**Results:**

Four (27%) out of 15 patients completed 33% of the recommended number of sets. For the total sample, the average percent of performed sets was 23% and for those who actually trained (*n* = 12) 26%. Patients and staff expressed a general positive attitude towards the unsupervised training as an addition to the supervised training sessions. However, barriers were also described—especially constant interruptions.

**Conclusions:**

Based on the predefined criterion for feasibility, the unsupervised training was not feasible, although the criterion was almost met. The patients and staff mainly expressed positive attitudes towards the unsupervised training. As even a small training dosage has been shown to improve the physical performance of geriatric inpatients, the proposed intervention might be relevant if the interruptions are decreased in future large-scale trials and if the adherence is increased.

**Trial registration:**

ClinicalTrials.gov: NCT02702557, February 29, 2016. Data Protection Agency: 2016-42, February 25, 2016. Ethics Committee: No registration needed, December 8, 2015 (e-mail correspondence).

**Electronic supplementary material:**

The online version of this article (10.1186/s40814-017-0202-3) contains supplementary material, which is available to authorized users.

## Background

Frailty is a state of increased vulnerability to poor resolution of homeostasis following a stress, which increases the risk of adverse outcomes including falls, delirium, and disability [1]. Aside from frailty being considered to confer a high risk of adverse outcomes, it also increases the risk of (re)hospitalization, institutionalization, and mortality [2, 3]. It affects 5–58% of the geriatric population [4], which is why effective rehabilitative strategies are warranted.

Sarcopenia is considered an important factor of frailty and is defined as a condition characterized by loss of muscle mass combined with decreased strength and physical performance [5]. It can be treated with strength training [6], which is why strength training for the older inpatient is a common prescription during hospitalization. Numerous studies have found that strength training is an effective treatment for frail geriatric inpatients [7–11] and can increase strength by 113% when performing high-intensity progressive resistance training for hip and knee extensors three times per week [11]. However, it is not always possible to ensure an adequate amount of supervised training needed for frail geriatric inpatients to minimize their loss of functioning [12]. This is due to a lack of resources among staff and lack of motivation on the part of the inpatient at the specific time of the supervised training session [13]. A possible solution to the problem could therefore be to supplement the supervised training sessions with feasible unsupervised exercises. In a recent meta-analysis, it has been shown that strength training with lower loads until failure seems to induce a similar muscle hypertrophy compared to higher loads [14]. Additionally, this tendency is also seen in the context of older adults [15]. Based on this, elastic band exercises might be a useful method.

Until now, the exact training dosage of unsupervised training has been unknown because the training dosage has been based on self-report measures resulting in both over- and underestimation [16, 17]. A new exercise-integrated technology, BandCizer Datalogger version 1 (BandCizer ®, DK) (subsequently referred to as BandCizer), makes it possible to objectively monitor the training dosage during unsupervised elastic band exercises, having received initial exercise instructions. This version of the BandCizer has never previously been tested in a complex and highly specialized hospital setting for frail geriatric inpatients. Before a future randomized controlled trial examining the potential effect of combining supervised and unsupervised strength training during hospitalization is performed, it is recommended to conduct a feasibility trial to inform a future large-scale study [18]. Therefore, the purpose of this study was to investigate the feasibility and acceptability of an unsupervised progressive strength training intervention monitored by the BandCizer for frail geriatric inpatients.

## Methods

A feasibility trial was designed to objectively investigate whether unsupervised elastic band exercises could be performed by a group of frail geriatric inpatients and to investigate how the intervention was accepted and experienced by patients and staff. The study was further designed to inform a future large-scale randomized trial on the effect of supplementing supervised physiotherapy sessions with unsupervised elastic band exercises.

### Ethics

The Ethics Committee of North Denmark Region assessed the feasibility trial and stated that no approval was required (December 8, 2015, e-mail correspondence, Ethical Committee of North Denmark Region). The journal is provided with the evidence. The Declaration of Helsinki was followed, and all patients gave written informed consent.

### Setting

Hospitalized patients were consecutively recruited from a geriatric ward at Aalborg University Hospital, North Denmark Region, Denmark, between the 29th of February and the 14th of April 2016.

### In- and exclusion criteria

Patients were included if they were at least 65 years of age, frail based on a score of at least 5/15 points on the Tilburg Frailty Indicator (TFI) questionnaire [19, 20], and if they were able to read and understand Danish. Patients were excluded if they had a low cognitive level defined as a score < 5/10 on the Short Portable Mental Status Questionnaire (SPMSQ) [21], if there were any contraindications to exercise (decision made by a medical doctor), and if the patients had a pacemaker (due to the possible influence of the magnet in the BandCizer).

### The unsupervised strength training intervention measured by the BandCizer

The intervention was described in accordance with the Template for Intervention Description and Replication (TIDieR) checklist guide [22] (Table 1) and the mechano-biological descriptors of resistance exercise stimuli described by Toigo and Boutellier [23] (Table 2).

**Table 1:** Template for Intervention Description and Replication (TiDieR) Items 1–12

Item	Description
Item 1: Brief name	Unsupervised Elastic Band Exercises for Frail Geriatric Inpatients
Item 2: Why	To increase the physical activity, muscle strength and physical performance of frail geriatric inpatients.
Item 3: What (materials)	• BandCizer Datalogger version 1 • 2.5 m Latex-free Elastic Bands in five possible loads (TheraBand). • Information about the benefits of staying active during hospitalization.
Item 4: What (procedures)	• Unsupervised elastic band exercises as a supplement to the standard supervised and physiotherapeutic training. • Instruction in elastic band exercises for both upper and lower extremity. • Information about the benefits of staying active during hospitalization. • Tests at baseline (at the time of hospitalization) and discharge: 30-s chair-stand test (STS) [29], De Morton Mobility Index (DEMMI) [30] and Barthel-100 Index [28].
Item 5: Who provided	• A project physiotherapist with five years’ clinical experience and experience with frail geriatric inpatients (first author, CRR) gave information and instructions to the patients. • Before the beginning of the intervention the project physiotherapist undertook a further 20 hours of training of the intervention itself. • A physiotherapist from the daily staff tested the patients at hospitalization and at discharge, and a nurse from the daily staff tested the patients with Barthel-100 Index.
Item 6: How	• Information to the patients was delivered in person. • Individual exercise instruction, 30 minutes duration (Day 1). • Follow-up on the execution of exercises (Day 2 and Day 4) if the patient was still hospitalized.
Item 7: Where	The intervention was carried out in the hospital room of each patient (bed and chair available) on the geriatric ward. The geriatric ward receives patients with acute illnesses assessed to have a rehabilitative potential.
Item 8: When and how much	• Instruction in executing unsupervised elastic band exercises with a BandCizer mounted to the elastic band. • Instruction in one exercise for the upper extremity and one exercise for the lower extremity. • The unsupervised elastic band exercises were recommended one time per day every day and for as long as the patient was hospitalized. • Three times ten repetitions (10–12RM) [7] according to a time-under-tension of 3 seconds for the concentric phase, 2 seconds for the isometric phase and 3 seconds for the eccentric phase (in total 240 seconds for the upper extremity and 240 seconds per leg for unilateral lower extremity exercises).
Item 9: Tailoring	Patients were tested at baseline to determine which type of elastic band they could execute the exercise with and maintain 10–12RM. The starting position was also defined at baseline. The exercises were continuously adapted to the patients during the course of the intervention if necessary.
Item 10: Modifications	Patients were only instructed in one exercise if any contraindications existed. E.g. a patient might have been instructed in an upper extremity exercise immediately after lower extremity surgery.
Item 11: How well (planned)	The patient was encouraged to execute the intervention and the rationale for the exercises was made clear to the patient. This information was verbally explained to the patient at baseline and follow-up sessions. In addition, a written note was handed to the patient where the information could be retrieved and the individual exercises were further described visually and linguistically.
Item 12: How well (actual)	The objectively monitored adherence to the exercises was part of the purpose of this study and described in detail elsewhere.

**Table 2:** Mechano-biological descriptors of resistance exercise stimuli

	LE level 1	LE level 2	LE level 3	UE level 1	UE level 2	UE level 3
Load magnitude	10–12 RM	10–12 RM	10–12 RM	10–12 RM	10–12 RM	10–12 RM
Number of repetitions	10	10	10	10	10	10
Number of sets	3/leg	3/leg	3	3	3	3
Rest in between sets (minutes)	2	2	2	2	2	2
Number of exercise interventions (days)	Every day	Every day	Every day	Every day	Every day	Every day
Duration of the experimental period (days)	During hospitalization	During hospitalization	During hospitalization	During hospitalization	During hospitalization	During hospitalization
Fractional and temporal distribution of the contraction modes per repetition and duration (seconds) of one repetition	Concentric: 3 s Isometric: 2 s Eccentric: 3 s	Concentric: 3 s Isometric: 2 s Eccentric: 3 s	Concentric: 3 s Isometric: 2 s Eccentric: 3 s	Concentric: 3 s Isometric: 2 s Eccentric: 3 s	Concentric: 3 s Isometric: 2 s Eccentric: 3 s	Concentric: 3 s Isometric: 2 s Eccentric: 3 s
Rest in between repetitions (seconds)	No	No	No	No	No	No
Time-under- tension (seconds)	8 s/rep 80 s/set 420 s/total/leg	8 s/rep 80 s/set 420 s/total/leg	8 s/rep 80 s/set 420 s/total	8 s/rep 80 s/set 840 s/total	8 s/rep 80 s/set 840 s/total	8 s/rep 80 s/set 420 s/total
Volitional muscular failure	Yes	Yes	Yes	Yes	Yes	Yes
Range of motion	Knee 0°–90° flexion	Knee 0°–90° flexion	Knee 0°–90° flexion	Elbow 0°–180° flexion	Elbow 0°–180° flexion	Elbow 0°–180° flexion
Recovery time in between exercise sessions (hours)	24 h	24 h	24 h	24 h	24 h	24 h
Anatomical definition of the exercise (exercise form)	Supine in bed. Elastic band under foot, arms fixated across chest. A knee extension is executed with first one then the other leg.	Sitting on a chair. Elastic band under foot, arms fixated by the armrest. A knee extension is executed with first one then the other leg.	Standing with hip width between the legs. Elastic band under both feet. Elastic band is held stretched with the arms across the chest. A chair is placed behind the patient. The patient gets up and sits down without touching the seat.	Supine in bed. Elastic band around headboard. The elastic band is held with both hands and pulled with the arms from a position in front of the body till behind the body.	Sitting on a chair with face against the headboard. Elastic band around headboard. Patient holds the elastic band with both hands and pulls the elastic band with the arms from a position in front of the body till behind the body.	Standing with the face against the headboard. Elastic band around headboard. Patient holds the elastic band with both hands and pulls the elastic band with the arms from a position in front of the body till behind the body.

Left row: The 13 mechano-biological descriptors of resistance exercise stimuli. The six right-sided rows: The description of the lower extremity (LE) and upper extremity exercises (UE) divided into three levels of progression [23]

**Fig. 1:**
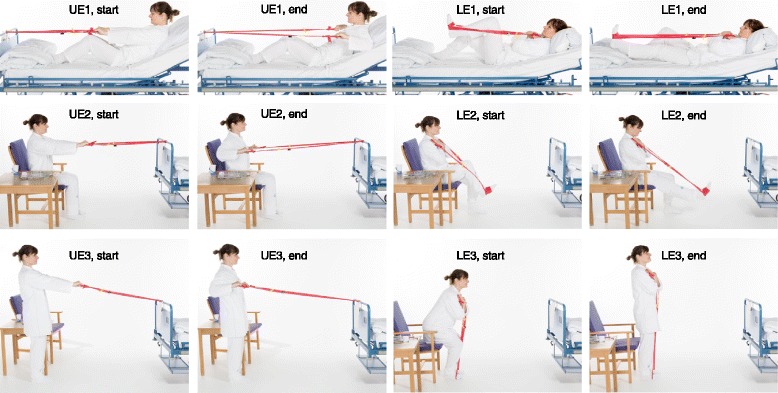
Elastic band exercises divided into three levels of progression. Elastic band exercises with start (start) and end position (end) shown for the three levels (1–3, 3 = highest level) of progression for the upper extremity (UE) and for the lower extremity (LE)

**Fig. 2:**
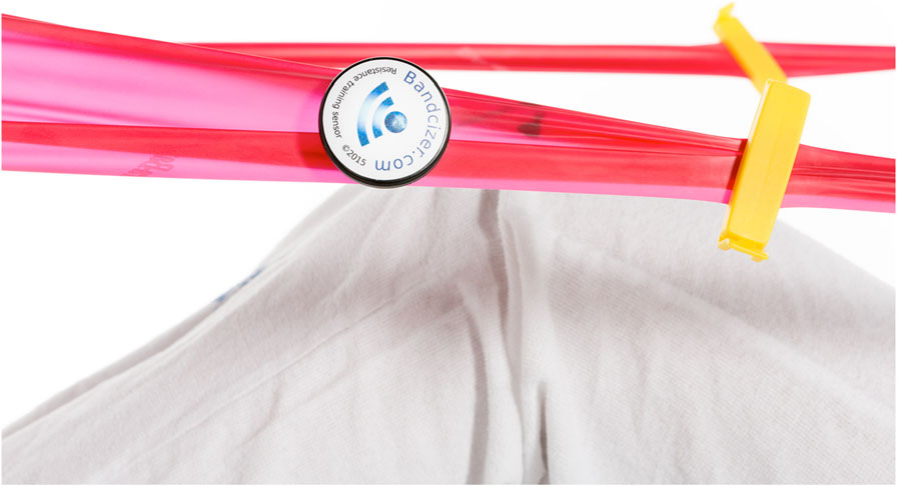
Mounting of the BandCizer on the elastic exercise band. The BandCizer mounted on the elastic exercise band at a distance of 5 cm from the handle marked by the attached plastic clips [27]

Patients were instructed in performing two unsupervised elastic band exercises (latex-free elastic exercise band, TheraBand™, The Hygenic Corporation and Performance Health, LLC, Canada) each day during the entire period of hospitalization, having received a thorough initial exercise instruction, an information sheet at the day of inclusion as well as follow-up instructions at days 2 and 4, if still hospitalized (Table 2). The follow-up instructions had a duration of a maximum of 15 min. The exercises included one exercise for the upper body and upper extremity and one for the lower extremity to target larger muscle groups and were divided into three levels of progression with different start positions (lying, sitting, standing) (Fig. 1) (Table 2) depending on the functional level of the frail geriatric inpatient. Each exercise was prescribed as three sets of 10 repetitions (10–12 repetition maximum (RM)) [7] each day and at any time of day. The sets were prescribed with a separation of 2-min pauses [24, 25]. The repetitions had a time-under-tension of 8 s (3 s for the concentric phase, 2 s for the quasi-isometric phase, and 3 s for the eccentric phase) [26] (Table 2). A BandCizer Datalogger version 1 was mounted on the elastic exercise band at a distance of 5 cm (measured with no tension of the elastic band) from the handle marked by the attached plastic clips [27] (Fig. 2).

### Data collection and analysis

The following patient data were collected: demographic (age and gender) and descriptive data (score of Barthel-100 Index [28], 30-s chair-stand test [29], and De Morton Mobility Index [30]).

The following data were collected from the BandCizer: number of sets, number of repetitions, average time-under-tension, and total time-under-tension. The data were calculated by an algorithm in the BandCizer Backend software (BandCizer ®, DK). The algorithm has been described in detail previously by Rathleff et al. [31]. In brief, a set was registered when a pause between repetitions > 1 min was present [24]. A repetition was based on a concentric, quasi-isometric and eccentric phase of the stretch signal as defined by Rathleff et al. [32]. To determine the time-under-tension for a single repetition, individual stretches were identified and counted by peak-detection and thresholding of the data, the relaxation level between stretches is identified as the minimal tension occurring between peaks, and the threshold for tension is chosen as 10% above the relaxed state with respect to the peak tension; the time-under-tension of each stretch is measured as the time where the tension is above this threshold. The total time-under-tension was defined as the total time of all repetitions in a single training set [33].

The predefined criterion for the intervention to be considered feasible for the patient group was that 33% of the prescribed number of sets per exercise (meaning at least one set of each prescribed exercise per day) should be performed by at least 30% of the included patients. The 33% of the prescribed number of sets was chosen because the execution of one set was seen as the initiation of a training session, because performance of a single set has shown to improve physical performance and muscle strength for older women [34]. Furthermore, this was considered the minimum limit of the exercise dose. The 30% adherence criterion was based on the adherence rates from previous studies ranging from 10 to 85% depending on the patient group, the illness, or definition used [35, 36] and because adherence to physical activity is reported to be as low as 30% in frail geriatric inpatients [37].

Descriptive methods were used to present the data. Mean (sd) was used to summarize continuous measurements and median (IQR) to summarize not normally distributed data.

The patient data were managed in Excel and IBM SPSS Statistics (version 23).

Data from the initial instruction were excluded from the analysis, as these data were from supervised training sessions and therefore not initiated by the patients themselves.

No formal sample size calculation was performed due to the descriptive character of the study and no efficacy testing was to be performed [38]. Approaches to sample size justification for pilot and feasibility trials vary greatly [39]. We aimed for a target sample size of 10–20 based on Julious [40], who recommends a sample size of 12 (per group) as a rule of thumb for a pilot study, and based on a 7-week inclusion period. If 12 participants were included before the 7-week inclusion period ended, we would continue to recruit until it ended or until 20 participants were included.

### Patient and staff interviews

The reporting of the qualitative part of the study was in accordance with the Consolidated Criteria for Reporting Qualitative Research (COREQ) [41].

**Fig. 3:**
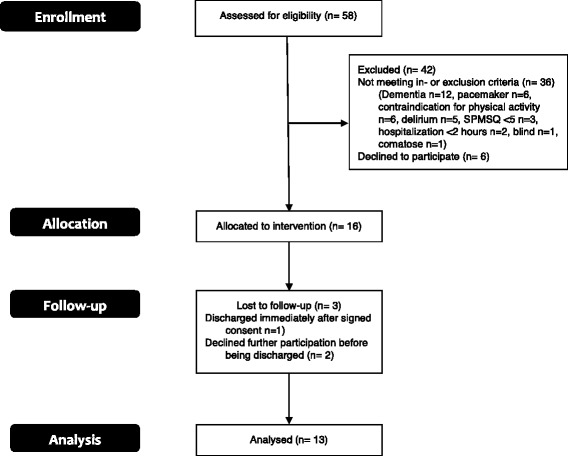
Patient flow diagram

Patient interviews: The day before patients were discharged from the hospital, a semi-structured interview was conducted at the bedside of each patient, there were thus other patients or staff sometimes present while conducting the interviews. Thirteen out of the 15 patients included in the study were interviewed (Fig. 3), and the interviews lasted 8–24 min. After the conducting of nine interviews, no new themes emerged, which indicated that data saturation was seemingly achieved [42]. The patients did not have the transcripts of their interview returned. The semi-structured interview guide was pilot-tested in three geriatric persons with no subsequent changes to the guide, other than the addition of an extra question (Table 3).

**Table 3:** Interview questions from the interview guide for patients and staff

Question number	Interview question, patients	Interview question, staff
1	How did you experience the training with the elastic band?	How did you experience the unsupervised training of the patients?
2	What challenges have you met during the course of your training with the elastic band?	What challenges do you think the unsupervised training present for the hospitalized patients?
3	What good things can you mention from training with the elastic band?	What advantages do you think the unsupervised training present for the hospitalized patients?
4	Was there anything that you would have wished was different?	Do you think that the unsupervised training can be conducted by your patients?
5	What makes you do the unsupervised training?	Have there been any surprises to you during the course of this study where the patients have been doing unsupervised training sessions?
6	What makes you not want to do the unsupervised training?	Are there any other things that you would like to add in the context of this study?
7	Could you have done more to execute the unsupervised training?	
8	Could I have done more to make you execute more of the unsupervised training?	
9	Do you think it has had an impact on your amount of training that there has been an eye kept on your amount of training?	
10	How often would you say that you have been doing the unsupervised elastic band exercises?	
11	Are there any other things that you would like to add?	

Staff interview: After the intervention period, the ward staff were interviewed. The focus group interview was semi-structured and held in a room away from the hospital ward. The four informants were a medical doctor (male, 30 years old, seniority on the geriatric ward: 6 months), a nurse (female, 31 years old, seniority on the geriatric ward: 6 years), an occupational therapist (female, 62 years old, seniority on the geriatric ward: 19 years), and a physiotherapist (female, 26 years old, seniority on the geriatric ward: 6 months). The interview lasted 39 min. The semi-structured interview guide was pilot-tested in a physiotherapist and a nurse and did not result in any subsequent changes (Table 3). The staff had the transcript of the interview returned, and nobody returned any comments or corrections.

All interviews were performed by the first author (CRR, BPhty, M.Sc.) who also instructed the patients in the execution of the exercises. The interviewer was categorized as moderately experienced and was present in the geriatric ward during the duration of the study, establishing a good acquaintance with the staff and the frail geriatric inpatients. The interviews were audiotaped, transcribed verbatim immediately after data collection [42] and analyzed in cooperation with the last author. A four-step data controlled analytic approach called systematic text condensation was used: (1) identification of themes from the transcription, (2) coding of meaningful units under themes, (3) subdivision of codes and forming of artificial citations, and (4) description of contents [42]. This procedure ensured that themes were derived from the data. No software was used in the coding process. Instead, a physical and material method was used by marking the single meaningful units and cutting them out of the raw and unprocessed material. Due to anonymity, ID numbers were used for patients and numbers for staff.

## Results and findings

### Recruitment

Fifty-eight patients were assessed for eligibility (Fig. 3) [43]. Of these, 36 were excluded based on the in- or exclusion criteria, mainly due to dementia. Twenty-two patients were eligible for enrolment but six did not want to participate. Sixteen patients were allocated to the intervention, and one patient was lost to follow-up because of the patient being discharged immediately after having signed the informed consent form. Fifteen patients were analyzed; however, two of these patients declined further participation before being discharged, which is why they were not interviewed.

### Demographics

The demographics and baseline characteristics of the patients are presented in Table 4. The average age was 86 years (SD 7.53). The patients scored 8 (SD 1.53) on the Short Portable Mental Status Questionnaire, 7 (SD 2.03) on the Tilburg Frailty Indicator, and had a Barthel-100 Index score at hospitalization of 60 (SD 28.03). Only a few patients had the De Morton Mobility Index and 30-s chair-stand test score recorded due to lack of ability to perform the test, which is why this average calculation was not performed.

**Table 4:** Demographics and baseline characteristics

ID	Gender	Age (years)	Diagnosis	SPMSQ (score)	TFI (score)	DEMMI (raw score)	STS (number)	Barthel-100 (score)
1	Woman	87	Fracture	10	8	N/A	N/A	73
2	Woman	93	Fracture	9	5	N/A	N/A	25
3	Man	91	Pneumonia	10	10	9	5	61
4	Man	93	Fracture	9	6	N/A	N/A	84
7	Woman	79	Pneumonia	9	5	18	10	90
8	Woman	90	Pneumonia	5	6	N/A	N/A	96
9	Woman	92	Fracture	7	8	N/A	N/A	24
10	Man	89	UTI	9	11	N/A	N/A	13
11	Man	71	Fracture	8	7	N/A	N/A	63
12	Woman	84	UTI	6	9	6	3	69
16	Woman	94	↓GC	7	9	N/A	N/A	68
18	Woman	82	Back pain	10	6	N/A	N/A	91
21	Man	75	COPD	7	5	N/A	N/A	49
24	Woman	77	↓GC	8	10	2	0	18
25	Woman	93	Dehydration	7	6	19	9	78

*SPMSQ*, Short Portable Mental Status Questionnaire, *TFI* Tilburg Frailty Indicator, *DEMMI* De Morton Mobility Index Score, *STS* 30 s chair-stand test, *Barthel-100* Index at the time of hospitalization, *UTI* urinary tract infection, *COPD* chronic obstructive pulmonary disease, *↓GC* decreased general condition, *N/A* not applicable

### Performance of elastic band exercises

**Fig. 4:**
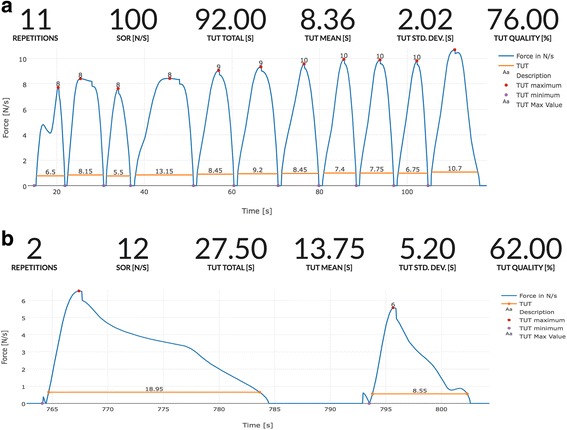
Two examples of individual training data. One exercise set performed close to that prescribed (**a**) and one very far from that prescribed (**b**). The blue curve indicates the force exerted during single repetitions. The time-under-tension (TUT) for each repetition is marked by the horizontal orange lines. Above the traces, a summary of the data from the BandCizer is shown. In the top panel, exercises performed close to that prescribed are shown. The 11 repetitions are close to the recommended 10 repetitions per set (repetitions). Likewise, the average time-under-tension is close to the prescribed 8 s per repetition (TUT mean (second)). In the lower panel, a performance far from that prescribed is shown. The two repetitions are far from the prescribed 10 repetitions per set (repetitions). Likewise, the average time-under-tension is far from the prescribed 8 s per repetition (TUT mean (second)). (Screendump from BandCizer Backend)

The patients varied greatly in how they performed the exercises, which is illustrated in Fig. 4 with two individual examples of training data (Additional file 1).

The training data for each patient is shown in Table 5. Four out of the 15 patients (27%) had performed at least 33% of the prescribed number of sets. The patients who did perform the exercises (*n* = 12) performed a median of 19.5% (IQR = 26.4%) of the recommended number of sets, 26.8% (IQR = 31.0%) of the recommended number of repetitions, and 27.4% (IQR = 38.0%) of the recommended total time-under-tension.

**Table 5:** Summarized training data for individual patients

ID	Exercise level	Elastic color	Actual/possible training days	Set (number)	Set (% prescribed)	Repetitions (number)	Repetitions (range/set)	Repetitions (%)	Total TUT (secon)	Total TUT (%)	Mean TUT (second)	std TUT (second)	Mean TUT deviation (second)
1	UE, lev1 LE, lev1—unilat.	Yellow	4/11	21	31.82	189	1–39	28.64	1967.60	37.26	11.20	1.87	3.19
2	UE, lev1 LE, lev1—unilat.	Red	4/4	9	37.50	375	10–89	156.25	2632.10	137.00	7.48	2.92	0.52
3	UE, lev2 LE, lev2	Red	3/4	5	13.89	118	10–40	32.78	890.57	30.93	7.40	2.36	0.60
4	UE, lev1 LE, lev1—unilat.	Green	1/2	1	08.33	23	23	19.17	229.20	23.88	8.35	1.66	0.35
7	UE, lev2 LE, lev3	Red	1/1	3	50.00	15	1–11	25.00	83.75	17.45	11.34	0.50	3.34
8	UE, lev2 LE, lev2	Yellow	1/6	1	01.85	4	4	0.74	10.80	0.25	2.70	1.11	5.30
9	UE, lev1 LE, lev1	Red	0/4	0	0.00	0	N/A	0.00	0	0.00	N/A	N/A	N/A
10	UE, lev1	Red	0/6	0	0.00	0	N/A	0.00	0	0.00	N/A	N/A	N/A
11	UE, lev1	Red	2/6	4	22.22	35	7–11	19.44	207.24	14.39	5.51	2.13	2.49
12	UE, lev2	Red	1/2	3	50.00	58	2–46	96.67	266.40	55.42	7.46	3.50	0.54
16	UE, lev1	Yellow	1/2	1	16.67	21	21	35.00	114.15	95.13	5.44	2.54	2.56
18	UE, lev1 LE, lev1	Yellow	1/2	3	16.67	24	2–13	13.33	204.30	14.19	9.46	2.38	1.46
21	UE, lev1	Red	0/1	0	0.00	0	N/A	0.00	0	0.00	N/A	N/A	N/A
24	UE, lev1	Red	2/7	3	14.29	55	3–32	26.19	355.70	21.17	5.91	1.50	2.09
25	UE, lev3	Green	6/6	9	50.00	266	21–40	147.78	783.05	54.38	3.10	0.79	4.90

Summarized training data for all patients, including an evaluation of feasibility for the patients individually. Percentages refer to the prescribed training dosage. All other numbers refer to the exact training dosage

*TUT* time-under-tension, *UE* upper extremity, *LE* lower extremity, *N/A* not applicable

### Findings from interviews

The three categories (codes) identified from the patient interviews were (1) *personal factors*, (2) *difficulties with the unsupervised training*, and (3) *positive effects of the unsupervised training*. From the staff interview, the three categories identified were (1) *optimism for unsupervised training*, (2) *requirements of the patients*, and (3) *organization of the daily schedule*—*a challenge*. From the united analysis of the two types of interviews, two themes arose (1) *advantages of unsupervised exercises* and (2) *challenges of unsupervised exercises* (Table 6).

**Table 6:** Main findings from interviews

	Patients (*N* = 13)	Staff (*N* = 4)
Advantages of unsupervised exercises (Based on patient interviews codes 1 and 3 and staff interview code 1)	(1) Good, simple and motivating (2) Help return to former level of function (3) Physical advantages (4) Good information (5) Enough help and follow-up (6) Flexible exercises (time of day) (7) Simple exercise tools	(1) Promote patient autonomy and responsibility (2) Increase communication about need of help in everyday activities (3) No negative aspects (4) Resource-light task (5) Great potential: (i) can lead to physical gain for patients; (ii) initiation of rehabilitation plans post-hospitalization possibly unnecessary (6) Flexible exercises (time of day)
Challenges of unsupervised exercises (Based on patient interviews code 2 and staff interview codes 2 and 3)	(1) Tiredness, pain, lack of desire to exercise, mind set on other things (2) Disturbances from staff, patients and relatives (3) Indifferent about number of repetitions (4) Need for the patient to independently mount the elastic band	(1) United responsibility among staff to keep focus on the performance of exercises (2) Information specifically to all professions about: (i) execution of the elastic band exercises; (ii) optimal place of documentation (3) Requirements for patients: (i) a certain cognitive level; (ii) motivation; (iii) ability to show initiative; (iv) quality in performance of exercises (4) Organization of the daily schedule

### Patients and staff were positive about the unsupervised training

In general, both patients and staff were positive about the unsupervised exercises. The personal factors influencing the patients’ attitudes towards the exercises and training dosage consisted of their thoughts about the exercises; their earlier experiences with executing training, motivation, mental factors; and their need for help to complete the exercises. The patients described the exercises as good, simple, and motivating, and, generally, the patients expressed a great satisfaction with the amount of follow-up and supervision of their exercises and did not express any difficulties in understanding and performing the exercises.

I have been very pleased with the elastic band exercises. I find them good and simple, anyone can perform them. I mean, you do not have to be a professor to work it out. I mean it is not difficult. There is no large instruction that you will have to figure out. You just have to know how the elastic bands have to be mounted and that it has to be done like this and that for it to be done the right way. That is actually it. I have been pleased with the training with the elastic bands. I think it is good and simple. (ID1, 106)

Yes. I think so (answer to the question: “Did you have all the help you needed?”). And if I needed help with anything I could just read it on the information sheet. So, I think it has been alright. *(*ID7, 30*)*

Some patients even found the exercises fun to execute, but the major cause of motivation for patients was that they thought that the exercises could help them return to their former level of functioning.

Well, for the time being I have only one goal after this unfortunate accident and that is to repeat everything once again but now for the opposite leg. I hope to just reach my former level, for the injured leg. I don’t expect to be walking around without my walker or anything. As long as it can become as it once was. (ID1, 76)

Like the patients, the staff were generally positive about the patients doing unsupervised elastic band exercises. They similarly found that the unsupervised exercises could lead to physical gain for the patients. At the same time, they found the unsupervised exercises to be a resource-light task containing great potential and with no negative aspects because there were no consequences if the exercises were initiated but not executed. One specific example of the potential the staff found in the unsupervised exercises was the probable opportunity for the exercises to replace further post-hospitalization rehabilitation.

Yes, it is a resource-light task, it is not as resource-heavy as, for instance, supervised physiotherapeutic training, which can be a great advantage. (Staff 1, 99)

I see nothing negative about it (the elastic band exercises), well, no I think they would be alright for those who are able to take an active part in their own training or for those who are able to perform the unsupervised training. For those, I only think it is a good initiative. (Staff 2, 46)

Besides the physical advantages, the staff found that the exercises could encourage the patients to take a greater responsibility for their own lives and found that the exercises could initiate communication with the patients about which everyday activities and displacements they needed help to perform and which ones they could perform independently.

Well, I think that it (doing the elastic band exercises) gives you some kind of responsibility, or whatever you say. Yes, well, in this context, that you yourself are responsible for getting better. (Staff 2, 97)

Moreover, both patients and staff mentioned organizational advantages of the unsupervised exercises. The exercises could easily be executed whenever possible during the day, which is why they were described as flexible. At the same time the patients found that the size of the equipment made it easy to access and to store within one’s own range, which gave the patient autonomy to execute the exercises whenever they wanted. In addition, the actual size of the equipment was an advantage, taking into account the cramped conditions in the geriatric ward.

### Organizational and individual issues challenging the unsupervised training

In both types of interviews, both organizational and individual challenges were highlighted. Almost every patient expressed the challenge of having the exercises fitted into a busy and unpredictable day in the geriatric ward. Further, the patients described their day as an endless series of disturbances from the staff, the other patients, and their own relatives.

Well, I simply got tired of people coming in every second minute. Like my son said: ‘It is like the Central Station of Fredericia (a city in Denmark)’. (ID16, 38)

The staff also mentioned that almost all activities in the geriatric ward were squeezed together in the first part of the day, which made the staff understand that the patients did not have time to do the exercises during the first part of the day. On the other hand, they thought that the patients should have time to do the exercises later in the day. In the geriatric ward, relatives were welcome during all times of the day. No specific hours were allocated for visits. The staff indicated that the daily schedule might need some changes.

First one patient has a visitor and then their neighbouring patient has yet another visitor, and so on, so then there is actually people in the room all the time. And it might be that the patient feels the need for that space to do their exercises. When their relatives are visiting, then they do not lie down and start to exercise, and some of them have very frequent visits. (Staff 1, 72)

The constant disturbances had a major impact on the training dosage. To some patients, the disturbances meant that the dosage became smaller than anticipated. To others, the disturbances meant that they gave up even initiating the execution of the exercises. Further, the statements about the training dosage indicated that the patients did not really care about the number of repetitions and sets but were more interested in whether they had been performing the exercises or not.

Counting, well, it has not really come so far but, well…I am doing the best I can. I do not quite know if I then repeat the exercises too many or too few times but that is also not really my concern. (ID2, 30)

Those patients who did experience issues with performing the exercises were primarily those who were not always able to independently mount the elastic band either because of pain or decreased mobility. In addition, some patients mentioned fatigue and decreased energy as individual challenges to performing the exercises. Yet other patients found that they themselves were the main obstacle and that they just had to pull themselves together or they were prevented from performing the elastic band exercises because their mind was set on different things outside the training itself. Different examples were given, such as fear of the future or fear of important answers in the context of treatment.

Well, it is when I am tired and I am not in the mood for anything and I find that everything has lost its meaning and I do not care if I make it or not. But what is constantly driving around in my head is that I am going home Tuesday (tomorrow) and even though I chose it myself – you heard that – I am afraid that it is too soon. (ID9, 49)

In accordance with the fact that not all patients felt the urge or energy to execute the unsupervised exercises, the staff identified different requirements of the patients to be able to execute the unsupervised exercises. They found that the patients needed to have a certain cognitive level to be able to perform the exercises on their own. The patients also needed motivation, the ability to show initiative and an acceptable level of performance of the exercises.

In general, I think that they (the elastic band exercises) impose some demands on our patients, where I find that not everyone would be able to participate – well, most patients would not be able to participate. That is what I am thinking at the moment. Compared to the other type of physiotherapy (supervised physiotherapy) I do not know whether there can be any challenges but at least they need to have a sufficient cognitive level to be able to join and to be part of it. (Staff 2, 37)

Besides the requirements for the patients to be able to execute the exercises, the staff highlighted three important factors for the exercises to be successful in the geriatric ward. The staff should have a united responsibility to keep focus on the performance of the exercises and to maintain the new initiative because this would likely increase the training dosage. Likewise, information about the execution of the elastic band exercises and the optimal place of documentation in the electronic patient record should be informed specifically to all relevant professionals.

… it is important to maintain that this is what we want and this is what we need to do, because I find that often when new initiatives are initiated they are up and running for the project period but…, and then ‘whew’. Off course small parts of the new initiatives linger but it is so easy to go back to the old routines again. (Staff 3, 147)

## Discussion

### Summary of primary results and findings

This seemingly is the first feasibility trial to objectively monitor training dosage during unsupervised elastic band exercises for frail geriatric inpatients in a complex and highly specialized hospital setting. Overall, the quantitative results showed that the intervention in its current form could not be deemed feasible. This was based on the predefined criterion of feasibility as too few patients reached the threshold, although the criterion was almost met. The qualitative findings based on the statements from patients and staff indicated that both advantages and challenges were related to the unsupervised elastic band exercises. However, both patients and staff mainly expressed positive attitudes towards the exercises.

### Feasibility of the intervention

The reason for the intervention not being feasible in its current form was that only four out of 15 patients (27%) executed a training dosage above the 33% criterion for number of sets for at least 30% of patients. The predefined feasibility criterion was based on previous adherence rates ranging from 10 to 85% [36, 37]. As this is the first study to objectively examine the adherence to elastic band exercises in frail geriatric inpatients, the feasibility criterion might initially have been too optimistic because no previous objectively measured rates could be used as reference. Adherence to supervised exercises typically exceeds that of unsupervised exercises [35, 36, 44, 45], and adherence rates based on self-report measures are often unrealistically high [16, 17, 46, 47]. Therefore, the feasibility criteria should perhaps have been set even lower because the patient group is in need of any intervention that can potentially encourage physical activity [2–4]. Yet, had only one more patient reached the exercise dosage threshold (lD1, Table 5, lacked only to have performed 1.2% of the prescribed number of sets) then the predefined feasibility criteria would have been met. In addition, the actual exercise dosage of the patients might be acceptable, as adherence to health interventions is considered a complex problem especially for individuals with chronic conditions [48], and a number of interdependent factors also influence the adherence to health interventions, including, among others, characteristics of the patient and the disease [49].

In the context of the patient characteristics, the staff mentioned factors of importance for the feasibility of the exercises. They mentioned that the exercises had built-in demands for the cognitive level of the patients, which the patients might not meet at all times during the hospitalization period. However, in this study, the Short Portable Mental Status Questionnaire was used as an inclusion test with a cut-off of five, which meant that only frail geriatric inpatients with no or only a mild cognitive impairment were included. The choice of excluding patients with dementia was made based on previous studies [11, 50]. Although the cognitive level of the frail geriatric patients seems to be an important factor for an intervention to be feasible [11, 50], inclusion of patients with no or mild cognitive impairment, as done in this study, was not enough to guarantee reaching the exercise dosage threshold.

Despite the intervention not being deemed feasible, based on the predefined quantitative criteria of feasibility, both patients and staff were positive about the unsupervised elastic band exercises. Some of the most frequent reasons mentioned for the positivity about the exercises were that they had an in-built possibility to regain an earlier functional level, they were flexible in time and task, and they gave the patients an increased responsibility for their own life. In addition, the implementation of exercises was thought of as a resource-light task for the staff and contains no risks. The positive view of the training did not seem to match the objective data. Therefore, both types of information are considered important to inform future studies as to how to adjust and improve the intervention before further testing is performed. Likewise, previous studies of physical activity interventions have advocated for further examination and adjustment of a given intervention, due to conflicting results based on different methodological approaches [51–53].

In combination with the positive attitude of patients and staff, the elastic band exercises could potentially prevent or decrease inactivity-related risks during hospitalization [54]. The reason for this being that the elastic band exercises can increase the amount of time the patient is active and not simply lying passively in the hospital bed. This seems to be important for frail geriatric inpatients, as previous studies, including a study from this particular geriatric ward [12], have shown that the patients had a very low physical activity level during hospitalization [12, 55]. Strength training has previously been shown to be a potent agent in the treatment of frail geriatric inpatients [7–10, 56–60]. In combination with the low occurrence of adverse events in both this and other training studies with this patient group [11, 58, 60, 61], the intervention should therefore be individualized and function as a potential supplement to the supervised physiotherapy sessions. This interpretation is based on the evidence from the literature showing the urgent need for strategies to improve the physical activity level of frail geriatric inpatients [2–4], and the diverse answers from patients regarding the sheer volume of factors influencing their exercise dosage. By individualizing and negotiating the goal of the training dosage with the individual patient, adherence has been shown to increase [44, 62]. Therefore, adherence to the exercises might be the issue that needs improving in order to increase the probability of the intervention being deemed feasible based on the quantitative criterion.

Based on this study, it cannot be decided whether the generally performed dosage of exercises confers a clinically meaningful benefit, but it has been shown that both a single set and multiple sets of similar exercises can improve the physical performance and muscle strength for older women [34], which is why a smaller exercise dosage might still be clinically meaningful in this group of frail geriatric inpatients. In addition, 12 out of 15 patients did perform some kind of exercises on their own that otherwise would not have been initiated. Coupled with the statements from the patients about the training dosage, in which they refer to being more interested in whether they had performed the exercises rather than caring about the number of repetitions and sets, it seems that the patients were actually interested in exercising. Furthermore, there were examples of two patients training persistently and more than prescribed (148 and 156% of the prescribed number of repetitions). A recent randomized controlled trial has shown that geriatric inpatients were able to increase their unsupervised physical activity level with the addition of feedback from an objective monitoring accelerometer [63]. Direct continuous feedback and specific times allocated for training may therefore be some of the future adjustments to be made to the intervention.

### Strengths and limitations

The external validity of the study findings might be limited based on the fact that only a single acute geriatric ward was used for the recruitment of patients and because only a few patients participated. However, it was strengthened by the data saturation that seemingly occurred after the execution of nine out of 13 patient interviews. Likewise, a large proportion of the included patients were interviewed as intended (87%), and the staff interviews included a representation of all professions working in the geriatric ward.

A limitation to consider is that cognitively impaired patients were excluded in this trial. This may have the consequence, that the feasibility actually is overestimated as a relatively large portion of geriatric inpatients in general are cognitively impaired. The time it took to recruit the 15 geriatric inpatients at a 10-bed geriatric ward may support this.

A desirability bias may potentially have occurred as the qualitative analysis was done by the person instructing the exercises. Given the nature of the questions, it is possible that the patients might have answered more positively than they actually wanted to. On the other hand, a more authentic answer might have been likely as they were familiar with the interviewer. In addition to this, the interviewer had been associated with the geriatric ward and the patients during a long period of time which has been previously highlighted as having a major positive impact on the quality of the interview [64].

It was considered a strength of this study that the adherence to the unsupervised elastic band exercises was objectively measured, but this made a direct comparison with the results of previous exercise studies on the patient group complicated, as these were primarily based on self-reports [58, 65, 66] or related to supervised exercises [8, 54, 56, 59, 66–69]. The quantitative and qualitative findings are considered strengths in relation to the design of future studies. The reason for this being that even though the quantitative results did not qualify the intervention, the qualitative findings can be used to improve the adaptation of the intervention to the specific population of frail geriatric inpatients, who are in urgent need of strategies to improve their physical activity level [2–4].

### Clinical implications for a future trial

Based on this study, no immediate clinical implications can be made, but indirect implications can be derived from it. In case of a future successful implementation of a modified version of the intervention in the context of hospitalization, it can be expected that the daily disturbances will be a challenge that should be managed. This could possibly be done by introducing designated times to complete the exercises. The type of intervention is considered to have in-built clinical implications because it is evaluated as being resource-light. A future large-scale randomized controlled trial might not be the obvious next step as achieving higher adherence is essential before designing and powering a future efficacy trial. Instead, the following step should be based on smaller studies examining whether adherence can be increased by context-specific and implementation-tailored adjustments of the intervention in relation to the registered advantages and challenges. A hybrid design study could involve the inpatients and relatives in developing an intervention that potentially increases the adherence [70].

## Conclusion

This is the first feasibility trial objectively monitoring training dosage during unsupervised elastic band exercises for frail geriatric inpatients. Based on the predefined criterion for feasibility, the unsupervised training was not feasible because only 27% of patients performed at least 33% of the prescribed number of sets, although the criterion of 30% was almost met. The patients and staff mainly expressed positive attitudes towards the unsupervised training. The most frequent reasons mentioned for the positivity about the exercises being that they had an in-built possibility to regain an earlier functional level, they were flexible in time and task, and they gave the patients an increased responsibility for their own life. In addition, the implementation of exercises was thought of as a resource-light task for the staff and contains no risks. However, despite the positive attitudes the exercises were not performed as much as defined in the feasibility criteria.

As even a small training dosage has been shown to improve the physical performance of geriatric inpatients, the proposed intervention might be relevant if the interruptions are decreased in future large-scale trials. However, before any efficacy trials are initiated, the adherence needs to be increased.

## Additional file

Additional file 1: BandCizer data. The training data of the individual patients from the BandCizer Backend. (XLSX 60 kb)

## Abbreviations

COPD: Chronic obstructive pulmonary disease
COREQ: Consolidated criteria for reporting qualitative research
DEMMI: De morton mobility index
IQR: Interquartile range
LE: Lower extremity
N/A: Not applicable
RM: Repetition maximum
SD: Standard deviation
SPMSQ: Short portable mental status questionnaire
STS: 30-s chair-stand test
TFI: Tilburg frailty indicator
TIDieR: Template for intervention description and replication
TUT: Time-under-tension
UE: Upper extremity
UTI: Urinary tract infection
